# Conicity Index and Other Anthropometric Indices in Relation to Visceral Fat Percentage in Adolescents with Overweight or Obesity: A Cross-Sectional Study

**DOI:** 10.7759/cureus.113293

**Published:** 2026-07-24

**Authors:** Kiran N Kudlikar, Vadde Y Reddy, Mohd Saeed Siddiqui, Priti Phatale, Avinash L Sangle, Madhurasree Nelanuthala, Surya Pratap Singh, Imtiyaz Ahmed, Doreswamy Chandranaik, Vandan R Bilala

**Affiliations:** 1 Pediatrics, Mahatma Gandhi Mission (MGM) Medical College, MGM Institute of Health Sciences, Aurangabad, IND; 2 Pediatrics, Samrat Endocrine Institute for Diabetes, Obesity and Thyroid, Aurangabad, IND

**Keywords:** adolescent obesity, body mass index (bmi), central obesity, conicity index, visceral adiposity, waist circumference (wc)

## Abstract

Background

Adolescent obesity is an increasing public health concern, particularly in South Asian populations where central adiposity and excess adiposity at relatively lower body mass index (BMI) are frequently observed. Visceral adiposity is a key determinant of cardiometabolic risk, but direct assessment using computed tomography or magnetic resonance imaging is impractical for routine clinical use. Anthropometric indices are therefore widely used as surrogate markers. The conicity index (CI), a composite measure incorporating waist circumference (WC), body weight, and height, has been proposed as an indicator of central fat distribution; however, its utility in overweight and obese adolescents remains uncertain. This study assessed the correlation between the CI and bioelectrical impedance analysis (BIA)-derived visceral fat percentage (VF%) in adolescents who are overweight and obese and compared its predictive performance with that of conventional anthropometric indices.

Methods

This hospital-based cross-sectional analytical study included 120 adolescents with overweight and obesity aged 10-19 years recruited by consecutive sampling from a tertiary care center in Maharashtra, India. Anthropometric indices, including BMI, WC, waist-to-hip ratio (WHR), waist-to-height ratio (WHtR), abdominal volume index (AVI), body adiposity index (BAI), and CI, were calculated using standard formulae. Body composition measurements, including VF%, were estimated using bioelectrical impedance analysis (Omron Karada Scan device, Omron Healthcare Co., Ltd., Kyoto, Japan). Because several variables were non-normally distributed, correlations were assessed using Spearman’s rank correlation, and hierarchical multiple linear regression was used to identify independent predictors of VF%.

Results

The median age of participants was 13.0 years (IQR 10.8-15.0), with a balanced sex distribution. WC and AVI showed the strongest correlations with VF% (ρ = 0.707 each, p < 0.001), followed by BMI (ρ = 0.696, p < 0.001). WHtR and WHR showed moderate correlations, whereas the CI demonstrated only a weak correlation with VF% (ρ = 0.267, p = 0.003). The CI was not significantly correlated with total body fat (BF%), subcutaneous fat (SCF), or fat mass index (FMI). In regression analysis, the base model including age, sex, and BMI explained 53.4% of the variance in VF%. Addition of WC significantly improved model performance (ΔR² = 0.091, p < 0.001; final R² = 0.624), whereas addition of the CI provided a smaller improvement (ΔR² = 0.049, p < 0.001). In the final model, BMI and WC were the only significant independent predictors.

Conclusion

The CI showed a statistically significant but weak correlation with VF% in this hospital-based cohort of adolescents who were overweight and obese, and its predictive performance was inferior to simpler measures, particularly BMI and WC. WC remains a practical and reliable anthropometric marker of central adiposity in this population. The CI may have a supplementary or research role but appears to provide limited additional value over conventional measures in this population.

## Introduction

Adolescence is a critical developmental period marked by rapid growth, pubertal change, and evolving lifestyle behaviors that influence long-term metabolic health [1]. Over recent decades, the prevalence of overweight and obesity among children and adolescents has risen substantially worldwide, with more than 340 million individuals aged five to 19 years affected globally by 2016 estimates [2]. India is experiencing a rapid nutritional and epidemiological transition, with urbanization, sedentary behavior, and dietary changes contributing to a dual burden of undernutrition and obesity. Indian studies report wide variation in adolescent overweight and obesity prevalence, with higher estimates generally observed in urban and higher socioeconomic groups [3,4].

Adolescent obesity is associated with early cardiometabolic abnormalities, including insulin resistance, dyslipidemia, hypertension, and clustering of cardiovascular risk factors [5,6]. Beyond overall adiposity, the distribution of adipose tissue is thought to be particularly relevant to this risk: visceral adipose tissue is metabolically active and has been linked, in other studies, to systemic inflammation, altered adipokine secretion, and insulin resistance [7-9]. The present study did not measure these downstream metabolic markers directly; rather, it examined visceral fat percentage as an anthropometric correlate, on the premise that accurate, non-invasive identification of visceral adiposity could support future risk-stratification efforts.

Body mass index (BMI) is the most widely used screening measure for overweight and obesity because it is simple, inexpensive, and feasible in clinical and community settings. However, BMI does not distinguish between fat mass and lean mass and provides limited information about body-fat distribution [10]. Adolescents with similar BMI values may have different degrees of visceral adiposity, particularly during periods of rapid growth and pubertal change [11]. Waist circumference (WC), waist-to-hip ratio (WHR), and waist-to-height ratio (WHtR) are commonly used alternatives that more directly reflect central adiposity and have been associated with cardiometabolic risk in pediatric and adult populations [12,13].

The conicity index (CI), introduced by Valdez, models the human body as a double cone and incorporates WC, body weight, and height into a single index of abdominal adiposity [14]. Adult studies have reported associations between the CI and cardiovascular risk, but its applicability in adolescents remains uncertain [15]. Theoretically, the CI could offer advantages over simpler anthropometric measures: by modeling the body as two conjoined cones, it is intended to capture abdominal shape independent of overall body size, and by incorporating weight and height alongside WC into a single dimensionless ratio, it aims to reflect the geometry of abdominal fat distribution rather than absolute girth alone [14]. We hypothesised that the CI would demonstrate a stronger association with visceral fat percentage than conventional anthropometric indices; whether this theoretical advantage holds in adolescents, who undergo rapid and heterogeneous changes in body composition, had not been adequately tested. This is important because adolescence is characterized by rapid and heterogeneous changes in body composition, fat distribution, and lean mass, all of which may affect the validity of anthropometric indices derived from adult geometric assumptions [16]. Despite increasing interest in composite anthropometric indices, evidence evaluating the CI specifically in overweight and obese adolescents remains limited, particularly in Indian clinical settings. The present study was therefore undertaken with two explicit aims: (i) to evaluate the correlation between the CI and bioelectrical impedance (BIA)-derived visceral fat percentage (VF%) among adolescents with overweight and obese, and (ii) to compare the CI’s predictive performance for VF% with that of conventional anthropometric measures, including BMI, WC, WHR, WHtR, abdominal volume index (AVI), and body adiposity index (BAI).

## Materials and methods

Study design and setting

This cross-sectional analytical study was conducted at a tertiary care hospital in Maharashtra, India. The primary objective was to evaluate the correlation between the CI and VF% among overweight and obese adolescents. Participants were recruited from outpatient services using consecutive sampling on all clinic days during the study period between April 2024 and May 2025.

Study participants

Adolescents aged 10-19 years who were classified as overweight or obese were eligible for inclusion. Participants were classified according to the WHO 2007 growth reference for school-aged children and adolescents [17], using BMI-for-age and sex z-scores; overweight was defined as BMI-for-age >+1 SD and obesity as >+2 SD relative to the WHO median [17]. Adolescents were excluded if they had chronic systemic illness such as renal, hepatic, or cardiac disease; endocrine disorders affecting growth or metabolism, including hypothyroidism or Cushing syndrome; acute illness at the time of assessment; use of medications known to alter body composition, such as systemic corticosteroids, anticonvulsants, or hormonal therapy; or restrictive dietary practices likely to influence body composition.

Sample size

The assumed correlation of ρ = 0.25 was selected a priori as a conservative, minimum clinically relevant effect size, consistent with Cohen’s small-to-moderate effect-size convention, in the absence of comparable pediatric literature relating the CI to BIA-derived visceral fat at the time of study design. Assuming this correlation, a two-tailed α of 0.05, and 80% power, the minimum required sample size was 120 participants. Sample size was calculated using G*Power software (version 3.1.9.7; Heinrich Heine University Düsseldorf, Germany). A total of 120 adolescents were included, and all completed the required assessments.

Anthropometric assessment

Anthropometric measurements were performed using standardized procedures. Body weight was measured to the nearest 0.1 kg using a calibrated digital weighing scale, with participants wearing light clothing and no footwear. Height was measured to the nearest 0.1 cm using a stadiometer, with participants standing erect in the Frankfort plane. WC was measured using a non-stretchable tape at the midpoint between the lower margin of the last palpable rib and the iliac crest at the end of normal expiration. Hip circumference was measured at the level of maximum protrusion of the buttocks. All measurements were obtained twice. A third measurement was taken if the difference exceeded the predefined acceptable range, and the mean of the two closest values was used for analysis.

Derived anthropometric indices were calculated as follows:

AVI 

\[ AVI = \frac{2 \times WC^{2} + 0.7 \times (WC - HC)^{2}}{1000} \] where WC and HC are measured in centimeters [18].

BAI

\[ BAI = \frac{HC}{\text{Height}^{1.5}} - 18 \] where HC is measured in centimeters and height in meters [19].

CI

\[ CI = \frac{WC}{0.109 \sqrt{\dfrac{\text{Weight}}{\text{Height}}}} \] as described by Valdez [14], where WC and height are measured in meters and weight in kilograms. 

Body composition assessment

Body-composition parameters including total body fat (BF%), subcutaneous fat (SCF), skeletal muscle mass (SMM), and VF% were estimated by BIA using the Omron Karada Scan HBF-3 device (Omron Healthcare, Inc., Kyoto, Japan) under standardized conditions, including avoidance of recent strenuous physical activity and ensuring adequate hydration. BIA is an indirect, algorithm-based method that does not measure visceral fat directly; computed tomography and magnetic resonance imaging remain the reference standards for visceral adiposity quantification, and BIA estimates may be influenced by hydration status, ethnicity, and device-specific prediction equations, which have been validated predominantly in adult populations [20].

Blood pressure assessment

Blood pressure was measured using a mercury sphygmomanometer with an appropriately sized cuff selected according to mid-arm circumference. Measurements were taken after at least five minutes of rest, with the participant seated and the arm supported at heart level. Two readings were recorded at an interval of two to three minutes. If the initial reading was elevated, a third reading was obtained, and the average of the last two readings was used [21].

Statistical analysis

Data were analyzed using jamovi software (version 2.4.1; The jamovi Project, Sydney, Australia). Continuous variables were summarized as median and interquartile range, and categorical variables as frequencies and percentages. Sex-based comparisons were performed using the Mann-Whitney U test for continuous variables and the chi-square test or Fisher’s exact test for categorical variables, as appropriate. Because several variables were not normally distributed, associations between anthropometric indices and body-composition parameters were assessed using Spearman’s rank correlation coefficient. Hierarchical multiple linear regression was used to identify independent predictors of visceral fat. Model performance was compared using R², adjusted R², Akaike information criterion, root mean square error, and change in R². A two-tailed p-value < 0.05 was considered statistically significant.

Ethical considerations

Ethical approval was obtained from the Ethics Committee for Research on Human Subjects, Mahatma Gandhi Mission (MGM) Medical College (Approval No.: MGM-ECRHS/2024/136, dated March 30^th^, 2024). Written informed consent was obtained from parents or legal guardians, and assent was obtained from adolescent participants.

## Results

Participant characteristics

A total of 120 adolescents with overweight and obesity were included, comprising 59 (49.2%) females and 61 (50.8%) males. The median age was 13.0 years (IQR 10.8-15.0 years), with females being significantly older than males (13.1 (11.2-16.4) vs. 12.0 (10.0-14.9) years; p = 0.029). The distribution of obesity categories was as follows: overweight, 41 (34.2%); and obese, 79 (65.8%), with no significant sex-based difference (p = 0.22). The median BMI was 27.1 kg/m² (IQR 23.7-30.9 kg/m²) and did not differ significantly between sexes (p = 0.426). Body composition analysis using BIA demonstrated significant sex-based differences in BF%, SCF, SMM, and fat mass index (FMI), with females exhibiting higher adiposity measures. However, VF% did not differ significantly between females and males (12.0 (10.0-17.0) vs. 12.0 (7.0-16.0); p = 0.274). Detailed baseline characteristics are presented in Table 1.

**Table 1:** Baseline demographic and anthropometric characteristics stratified by sex Data are presented as median (IQR) for continuous variables and n for categorical variables. Continuous variables are compared using the Mann-Whitney U test, and the corresponding statistic is the U value; categorical variables are compared using the chi-square test, and the corresponding statistic is the chi-square (χ^2^) value. Degree of freedom (df) for the obesity class is 2 and for SMR stage, 4. BMI: body mass index; WC: waist circumference; WHR: waist-to-hip ratio; WHtR: waist-to-height ratio; AVI: abdominal volume index; SCF: subcutaneous fat; SMM: skeletal muscle mass; FMI: fat mass index; SMR: sexual maturity rating; BP: blood pressure.

Variable	Female n = 59 (49.2 %)	Male n = 61 (50.8 %)	Total n = 120 (100%)	Statistic	p-value
Age (years)	13.1 (11.2 to 16.4)	12.0 (10.0 to 14.9)	13.0 (10.8 to 15.0)	1385	0.029
Weight (kg)	61.4 (49.9 to 72.5)	62.0 (46.4 to 76.0)	61.7 (48.0 to 75.2)	1764	0.852
Height (cm)	149.7 (141.9 to 155.5)	151.0 (138.2 to 162.0)	150.0 (140.0 to 160.4)	1664	0.477
BMI (kg/m^2^)	27.8 (23.6 to 31.8)	26.8 (23.7 to 30.4)	27.1 (23.7 to 30.9)	1648	0.426
Obesity Class n (%)					
Overweight	17 (28.8)	24 (39.3)	41 (34.2)	1.51	0.22
Obesity	42 (71.2)	37 (60.7)	79 (65.8)		
WC (cm)	90.0 (80.0 to 97.5)	86.0 (79.0 to 98.0)	88.0 (79.0 to 98.0)	1624	0.355
Hip Circumference (cm)	97.0 (84.0 to 110.0)	90.0 (84.0 to 105.0)	94.0 (84.0 to 108.2)	1571	0.231
WHR	0.9 (0.9 to 1.0)	0.9 (0.9 to 1.0)	0.9 (0.9 to 1.0)	1683	0.538
WHtR	0.6 (0.6 to 0.6)	0.6 (0.5 to 0.6)	0.6 (0.5 to 0.6)	1514	0.134
Conicity Index	1.3 (1.2 to 1.3)	1.3 (1.2 to 1.3)	1.3 (1.2 to 1.3)	1775	0.898
AVI	21.7 (17.2 to 25.5)	19.8 (16.8 to 25.8)	20.8 (16.8 to 25.8)	1631	0.375
Body Fat (%)	44.0 (38.0 to 48.9)	36.5 (28.6 to 41.0)	40.0 (33.8 to 45.3)	914	<0.001
SCF	42.7 (26.9 to 48.6)	26.0 (20.0 to 39.0)	34.5 (22.9 to 44.0)	1009	<0.001
SMM	49.0 (30.6 to 56.0)	30.6 (27.9 to 42.8)	37.0 (29.0 to 52.0)	956	<0.001
Visceral Fat (%)	12.0 (10.0 to 17.0)	12.0 (7.0 to 16.0)	12.0 (10.0 to 17.0)	1592	0.274
Fat Mass (kg)	26.3 (18.3 to 34.3)	20.8 (15.7 to 30.6)	22.8 (16.0 to 32.7)	1346	0.017
Fat Free Mass (kg)	35.5 (29.0 to 41.1)	40.3 (29.6 to 46.1)	36.8 (29.2 to 44.0)	1419	0.046
FMI	12.3 (9.2 to 14.8)	9.0 (7.5 to 12.3)	10.5 (8.1 to 13.8)	1149	0.001
SMR Stage					
0	7 (11.9)	19 (31.1)	26 (21.7)	11.46	0.012
1	16 (27.1)	20 (32.8)	36 (30.0)		
2	11 (18.6)	11 (18.0)	22 (18.3)		
3	15 (25.4)	9 (14.8)	24 (20.0)		
4	10 (16.9)	2 (3.3)	12 (10.0)		
Systolic BP (mmHg)	122.0 (114.0 to 127.0)	118.0 (110.0 to 128.0)	120.0 (112.0 to 128.0)	1695	0.58
Diastolic BP(mmHg)	80.0 (70.0 to 86.0)	80.0 (70.0 to 84.0)	80.0 (70.0 to 84.0)	1666	0.477

Correlation between anthropometric indices and body composition

**Figure 1:**
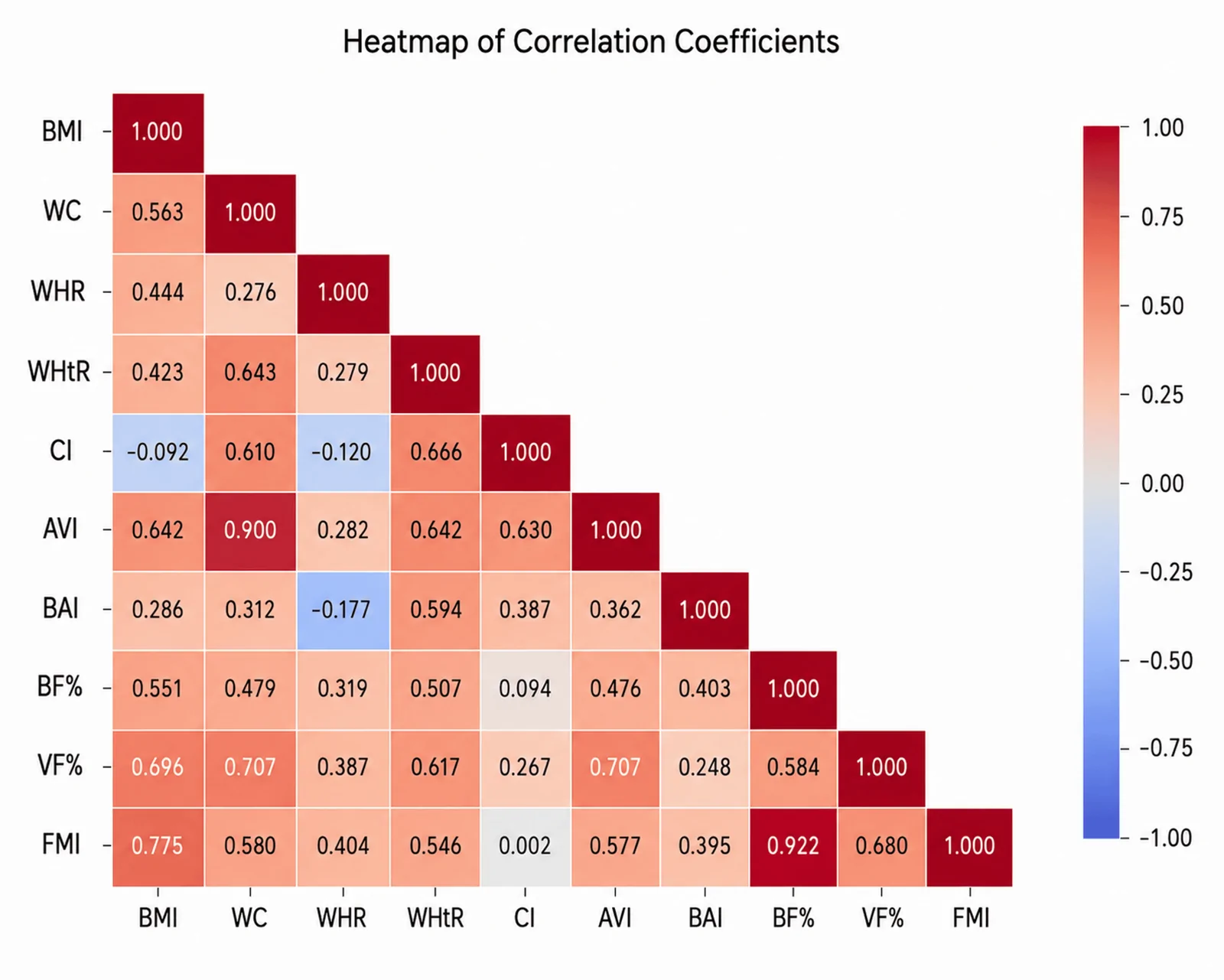
Heatmap of Spearman rank correlation coefficients Correlation coefficient values are Spearman's rho. BMI: body mass index; WC: waist circumference; WHR: waist-to-hip ratio; WHtR: waist-to-height ratio; AVI: abdominal volume index; SCF: subcutaneous fat; SMM: skeletal muscle mass; VF%: visceral fat percentage; FMI: fat mass index; BAI: body adiposity index; CI: conicity index

Spearman’s rank correlation analysis demonstrated significant positive associations between most anthropometric indices and VF%. WC and AVI showed the comparatively highest correlations with VF% among the indices studied (both ρ = 0.707, p < 0.001), a moderate-to-strong association, followed by BMI (ρ = 0.696, p < 0.001). Among derived indices, WHtR showed a moderate correlation (ρ = 0.417, p < 0.001), while WHR demonstrated a weaker but significant association (ρ = 0.387, p < 0.001). In contrast, the CI showed the weakest association with VF% among the indices studied (ρ = 0.267, p = 0.003). The CI did not correlate significantly with BF% (ρ = 0.094, p = 0.306) or FMI (ρ = 0.002, p = 0.985), suggesting limited ability to reflect overall adiposity. In contrast, FMI correlated strongly with BMI (ρ = 0.775, p < 0.001) and BF% (ρ = 0.922, p < 0.001). BF% also correlated significantly with BMI, WC, and WHR. The correlation matrix is presented in Figure 1.

Multiple linear regression analysis

Multiple linear regression models were developed to evaluate predictors of VF%. The base model, including age, sex, and BMI, explained 53.4% of the variance in visceral fat (R² = 0.534, adjusted R² = 0.521). The addition of WC significantly improved model performance (ΔR² = 0.091, p < 0.001), yielding a final R² of 0.624 and improved model fit. The AVI provided a comparable improvement (ΔR² = 0.087, p < 0.001), consistent with its strong mathematical dependence on WC. In comparison, the addition of the CI produced a smaller but statistically significant improvement relative to the base model (ΔR² = 0.049, p < 0.001). Other indices, including WHR, WHtR, and BAI, did not significantly enhance predictive performance. The most parsimonious clinically relevant model included age, sex, BMI, and WC. Model comparisons are presented in Table 2.

**Table 2:** Comparison of multiple linear regression models predicting visceral fat percentage R²: coefficient of determination; AIC: Akaike Information Criterion; RMSE: root mean square error; ΔR²: increase in explained variance compared with the base model. BMI: body mass index; WC: waist circumference; WHR: waist-to-hip ratio; WHtR: waist-to-height ratio; AVI: abdominal volume index; VF%: visceral fat percentage; BAI: body adiposity index; CI: conicity index; F: F-statistic value of the model; df: degrees of freedom.

Model	R	R²	Adjusted R²	AIC	RMSE	F	df1	df2	p value	ΔR²	p value (ΔR²)
Base (Age+Sex+BMI)	0.73	0.53	0.521	678	3.92	44	3	116	< .001	-	-
Base+WC	0.79	0.62	0.611	655	3.52	48	4	115	< .001	0.091	< .001
Base+WHR	0.73	0.54	0.519	680	3.91	33	4	115	< .001	0.002	0.529
Base+WHtR	0.74	0.54	0.527	678	3.88	34	4	115	< .001	0.009	0.134
Base+CI	0.76	0.58	0.568	667	3.71	40	4	115	< .001	0.049	< .001
Base+BAI	0.73	0.54	0.519	680	3.91	33	4	115	< .001	0.002	0.534
Base+AVI	0.79	0.62	0.607	656	3.54	47	4	115	< .001	0.087	< .001
Base+WC+CI	0.8	0.63	0.615	654	3.48	39	5	114	< .001	0.098	< .001
Base+WC+AVI	0.79	0.63	0.61	656	3.51	38	5	114	< .001	0.093	< .001

Regression coefficients of the final model

In the final model including age, sex, BMI, and WC, BMI was the strongest independent predictor of VF%, followed by waist circumference. The unstandardized coefficient for BMI was 0.499 (standardized β = 0.454, p < 0.001), and the unstandardized coefficient for WC was 0.188 (standardized β = 0.391, p < 0.001). Thus, each unit increase in BMI was associated with a 0.499-unit increase in VF%, and each centimeter increase in WC was associated with a 0.188-unit increase, independent of the other variables. Age and sex were not statistically significant predictors but were retained in the model due to biological relevance. The derived prediction equation was:

\[ \text{Visceral Fat (%)} = -19.879 + 0.499 \times BMI + 0.188 \times WC + 0.172 \times Age - 0.253 \times \text{Sex}_{\text{Male}} \]

Detailed regression coefficients are presented in Table 3.

**Table 3:** Final multiple linear regression model for prediction of visceral fat percentage Regression coefficients represent adjusted effects in the final model. BMI: body mass index; WC: waist circumference; SE: standard error of estimate; CI: confidence interval; t: t-statistic value.

Predictor	Estimate	SE	Lower 95% CI	Upper 95% CI	t value	p value	Stand. Estimate
Intercept	-19.879	2.6	-25.029	-14.728	-7.65	< .001	-
Age	0.172	0.156	-0.136	0.48	1.106	0.271	0.079
Sex: Male	-0.253	0.674	-1.588	1.083	-0.38	0.709	-0.043
BMI	0.499	0.076	0.348	0.649	6.577	< .001	0.454
WC (cm)	0.188	0.036	0.117	0.259	5.264	< .001	0.391

## Discussion

This study evaluated the relationship between the CI and BIA-derived VF% among adolescents with overweight and obesity and compared its performance with conventional anthropometric indices. The principal finding was that although the CI was statistically correlated with VF%, its correlation and predictive contribution were substantially weaker than those of WC, AVI, and BMI.

WC and AVI showed the strongest correlations with VF%, followed closely by BMI. This finding supports the established role of waist-based measures as practical markers of central adiposity [12]. WC directly reflects abdominal size and is therefore more closely related to visceral fat accumulation than composite indices that mathematically adjust for weight and height. Although BMI does not distinguish between fat and lean mass [10], it remained a strong predictor of VF% in this cohort of adolescents with overweight and obesity; one plausible, though not directly tested, explanation is that overall adiposity was uniformly high across this restricted sample.

Contrary to our a priori hypothesis, the CI did not outperform conventional indices; instead, it showed the weakest association with VF% among the indices examined. This weak correlation should not be interpreted as evidence that the CI lacks any clinical value; several, likely overlapping, explanations are plausible, including the indirect, algorithm-based nature of BIA-derived VF% relative to imaging methods, rapid and heterogeneous changes in body composition during adolescent growth and puberty, possible ethnic-specific patterns of fat distribution, and the restriction of this cohort to adolescents already classified as overweight or obese, which narrows the range of adiposity and may attenuate observed correlations. As a post hoc hypothesis rather than a directly tested finding, we suggest that the CI may capture body shape or abdominal geometry rather than true adiposity burden. Mechanistically, the CI’s comparatively weak performance may partly reflect its dependence on total body weight in its denominator: during puberty, fat and lean mass tend to increase together, which may dilute the specificity of a weight-normalized waist index for visceral fat, unlike WC, which reflects abdominal girth directly. The finding is also important because the CI was originally developed using an adult geometric model in which central fat accumulation changes the body shape from cylindrical to biconical [14]. Adolescents, however, undergo rapid and heterogeneous changes in height, weight, lean mass, and fat distribution, particularly during puberty [16]. These developmental changes may reduce the validity of adult-derived anthropometric assumptions in pediatric populations. Prior evaluations of WC, WHR, and the CI as screening tools for trunk fat mass in children and adolescents aged three to 19 years, using dual-energy X-ray absorptiometry as the reference method, similarly found that WC substantially outperformed the CI [22]. Likewise, a study of 314 children found that WHtR was a good predictor of excess body fat measured by BIA analysis, whereas the CI demonstrated poor predictive performance (area under the curve <0.70) [23]. Collectively, these studies indicate that the CI has generally been less accurate than WC or WHtR for identifying adiposity in children, although differences in age groups, adiposity measures, and study populations may contribute to variability in findings; future studies could usefully compare the CI directly against newer composite anthropometric indices in adolescent cohorts. Because this cohort was drawn exclusively from a single tertiary-care center and restricted to adolescents already classified as overweight or obese, these findings should not be generalized to unselected, community-based adolescent populations, in whom the correlation structure between the CI and visceral adiposity may differ.

The regression analysis further supports this interpretation. The base model containing age, sex, and BMI explained more than half of the variance in VF%. The addition of WC substantially improved model performance, whereas the addition of the CI provided a smaller improvement. Importantly, when WC was already included, the incremental gain from adding the CI was minimal. This indicates that the CI does not offer substantial additional clinical information beyond BMI and WC.

AVI performed similarly to WC, but this is expected because it is mathematically derived largely from WC [18]. Therefore, despite comparable statistical performance, AVI may not provide a practical advantage over the simpler measurement of WC. Similarly, WHtR, WHR, and BAI did not significantly improve prediction when BMI was already included in the regression model.

The findings have practical clinical implications. In resource-limited or routine clinical settings, BMI and WC remain simple, inexpensive, and reproducible tools for identifying adolescents with increased visceral adiposity [10,11]. WC, in particular, is easy to measure and directly reflects central adiposity [12]. The CI may be used as an adjunctive research measure, but the present findings do not support replacing conventional anthropometric measures with the CI in adolescent obesity assessment [22,23].

This study has several strengths. It focused specifically on adolescents who were overweight and obese, a clinically important group at increased risk for future cardiometabolic disease. It compared multiple anthropometric indices within the same cohort and used standardized measurement procedures.

However, several limitations should be acknowledged. First, the cross-sectional design precludes causal inference. Second, BIA is an indirect method and is less precise than reference imaging techniques such as computed tomography or magnetic resonance imaging [20]; body composition estimates were not validated against an imaging reference in this cohort, and the device’s prediction equations have been validated predominantly in adult populations. Third, the hospital-based sampling strategy may limit generalisability to community-based adolescent populations and may introduce selection bias; detailed participant-flow data (numbers screened, excluded, and refusal rate) were not systematically logged during recruitment and are not reported. Fourth, biochemical cardiometabolic markers such as fasting glucose, insulin resistance indices, and lipid profile were not included, limiting assessment of metabolic risk beyond anthropometry and body composition. Fifth, although sexual maturity rating was recorded, it was not entered as a covariate in the regression models, and pubertal stage may confound the relationship between anthropometric indices and visceral fat. Sixth, dietary intake, physical activity, and socioeconomic status-all of which influence body composition-were not assessed and could not be adjusted for. Seventh, formal multicollinearity diagnostics (e.g., variance inflation factors) and residual diagnostics (e.g., Cook’s distance, residual plots) were not performed for the regression models; given the correlation observed between WC, AVI, and BMI (Figure 1), this is a relevant caveat, and the model was not internally validated by bootstrapping or cross-validation, nor externally validated in an independent cohort, so its predictive performance should be interpreted as exploratory rather than confirmed. Eighth, formal intra-/inter-observer reliability statistics for repeated anthropometric measurements were not calculated. Finally, although the sample size was adequately powered for the primary correlation analysis, it was not calculated to support the secondary, sex-stratified comparisons reported in Table 1, which should therefore be interpreted with caution. Future research should include longitudinal, community-based cohorts with biochemical markers, pubertal-stage adjustment, and reference imaging methods to validate anthropometric indices across different stages of adolescent development. Establishing age-, sex-, and ethnicity-specific cut-offs for composite indices such as the CI may also help clarify whether they have clinical utility in pediatric populations.

## Conclusions

In this hospital-based cohort of adolescents with overweight and obesity, the CI showed a statistically significant but weak association with VF% and was inferior to BMI and WC in predicting visceral adiposity. WC showed the highest correlation with VF% among the indices examined and remains a practical and reliable marker of central adiposity in clinical settings. Although the CI incorporates multiple anthropometric parameters and has theoretical appeal, it provided limited additional value beyond conventional measures in this population. These findings apply specifically to adolescents already classified as overweight or obese in a single tertiary-care setting and should not be extrapolated to adolescents generally. Within this population, BMI and WC should remain the primary anthropometric tools for obesity and central adiposity assessment, while the CI may be considered only as a supplementary or research measure.
